# The Delivery of Diagnosis by Child Psychiatrists: Process Characteristics and Correlates of Distress

**DOI:** 10.3389/fpsyt.2021.632207

**Published:** 2021-03-22

**Authors:** Ayelet Brand-Gothelf, Ilanit Hasson-Ohayon, Nimrod Hertz-Palmor, Dana Basel, Doron Gothelf, Orit Karnieli-Miller

**Affiliations:** ^1^Sackler Faculty of Medicine, Tel Aviv University, Tel Aviv, Israel; ^2^Department of Psychology, Bar-Ilan University, Ramat Gan, Israel; ^3^Sheba Medical Center, Tel Hashomer, Israel; ^4^School of Psychological Sciences, Tel Aviv University, Tel Aviv, Israel

**Keywords:** diagnosis delivery, child psychiatrists, distress, schizoprenia, autism spectrum disorder, attention deficit hyperactivity disorder

## Abstract

We describe the attitudes of child psychiatrists toward diagnosis delivery (DD) and explore potential stressful factors associated with the process. Eighty Israeli child psychiatrists completed a questionnaire on their perceptions of DD of schizophrenia, autism spectrum disorder (ASD), and attention deficit/hyperactivity disorder (ADHD). We also conducted semi-structured in-depth interviews with 12 child psychiatrists who were asked to share their personal experience with DD. The questionnaire responses revealed that child psychiatrists perceived schizophrenia and ADHD as the most and least severe disorders, respectively, and its treatment as being ineffective and effective, respectively. They expressed negative perceptions toward DD of schizophrenia and positive perceptions toward DD of ADHD. The results of linear regressions revealed that some factors predicted distress accompanying DD in all three diagnoses, such as lack of professional experience, negative perceptions of DD, and the effect of parents' attitudes of opposition to the diagnosis. The interviews revealed that DD was often described by psychiatrists as an emotional experience and that the psychiatrists' age, and whether the psychiatrists identified more with the child or the parent, affected their attitude toward DD. Lastly, the psychiatrists expressed feelings of loneliness in the procedure of DD and their wish to share and reflect on their experiences with others. These findings may contribute to a better understanding of the clinically important topic of DD in child psychiatry that has not been adequately addressed and help deal with psychiatrists' challenges in this task.

## Introduction

The process of diagnosis delivery (DD), often involving the breaking of bad news to patients (1), includes stressful elements as physicians struggle with finding the best way to inform patients and relatives of a diagnosis that is perceived as having negative implications on various life domains (2). The physicians' perceptions of the effect of the diagnosis on the patient's life, and their attitudes specifically toward DD, were found to affect the nature of the disclosure of a diagnosis (3–5). A physician's lack of communication skills, which are essential in DD, may also increase the likelihood of the physician's experience of associated stress (2).

A few studies on physicians' experience with DD were conducted among samples that included participants from diverse medical fields. For example, Ptacek et al. (6) showed that ~18% of a mixed sample of physicians experienced stress in the process of DD, and 42% of them reported that it lasted from several hours to several days. However, the majority of the reviews, commentaries, and reports of empirical studies on DD are focused upon medical settings that are perceived as most challenging, such as oncology, pediatric, emergency settings, and neurology [e.g., (2, 5, 7–9)]. Those reports describe the struggle, distress, and often the actual avoidance of DD among physicians.

Studies on DD in psychiatry in general and specifically in child psychiatry are relatively rare. They describe unique characteristics due to two factors: the uncertainty of the accuracy of diagnosis due to the absence of objective tests and, even more so, the social stigma of mental illness (10), which may be present among professionals as well (11). For example, professionals working in psychiatry reportedly perceive psychiatric illness in a stigmatic way, viewing it as dangerous and as possessing extensive negative implications on potential career and/or social isolation (12). In addition, professionals working in psychiatry who do not hold stigmatic attitudes may nevertheless be sensitive to the potentially harmful implications of labeling and stigma on patients and their families and may therefore experience distress accompanying DD (13). While the internalization of stigma toward mental illness is evident not only among patients but also among their family members (14), child psychiatrists may struggle with the fear that parents will view the diagnosis in a stigmatic way.

The few studies, reviews, and commentaries that have addressed DD in psychiatry assessed it mainly from the perspective of the patients and families, the majority of whom learned about the diagnosis in the hospital discharge form or in an incidental encounter with a professional staff member (15). Others adopted a comprehensive approach and assessed patients, caregivers, and professionals' perspectives and focused on the experience of DD mostly among patients [e.g., (16)]. There is only one study that did explore adult psychiatrists' experiences of DD in the setting of schizophrenia and found that, overall, psychiatrists perceived disclosure as problematic, unproductive, and harmful (13). Data on DD in child psychiatry is even more sparse, and the limited studies that do exist focus mainly on parents' attitude to the delivery of an ASD diagnosis (17–19). To the best of our knowledge, there are no studies on the perceptions of DD among child psychiatrists. The goal of our study, therefore, was to better understand the perspectives of child psychiatrists in the process of DD. Our specific aims were two-fold. First, we wanted to explore factors that are related to the attitudes of child psychiatrists toward three different disorders and toward their DD. Specifically, we hypothesized that child psychiatrists will perceive schizophrenia as the most severe mental disorder, its treatment as relatively ineffective, express negative perceptions toward its DD, and experience its DD as distressing. Conversely, we hypothesized that they will perceive ADHD as the least severe disorder, its treatment as relatively effective, will have positive perceptions toward its DD, and will experience its DD as comparatively less distressing. As for ASD, we hypothesized that psychiatrists will perceive its level of severity as moderate, its treatment as ineffective, that they will have both positive and negative perceptions toward its DD, and that that they will experience its DD as moderately distressing. Secondly, we sought to identify factors associated with the distress of child psychiatrists associated with the DD process. We hypothesized that they would include lack of professional experience, negative perceptions of DD, and the perception of parents as being in opposition of the diagnosis. To further explore additional potential factors, a qualitative exploratory component was added.

## Methods

### Participants and Procedures

The participants were recruited to the current study during a conference of the Israel Child and Adolescent Psychiatry Association, which was held in January 2019. Seventy child psychiatrists who attended the conference were informed of the study's purposes and procedure and assured of anonymity, confidentiality, and their right to withdraw at any stage. After providing their written informed consent, they were asked to complete a self-report questionnaire on their positions and experiences regarding DD. An additional 40 child psychiatrists (listed in the Israeli Medical Association mailing list) who did not attend the conference received an email containing an invitation to participate, along with a link to a Google Forms online survey. All data for this survey were obtained between January 28, 2019, and February 14, 2019.

We also conducted semi-structured in-depth 1-h (average) phone interviews with 13 child psychiatrists to learn about their experiences and perceptions related to DD. Those interviewees were chosen from the child psychiatrists listed in the Israel Medical Association using a maximum variation sampling to represent a variety of professional experience, sex, and geographic distributions (20). All interviewees were contacted directly by telephone (by ABG) after they were informed of the rationale and procedure of the study, and assured of anonymity, confidentiality, and their right to discontinue the interview. Those who agreed to participate in the study gave their informed consent.

The Sackler School of Medicine Ethics Committee at the Tel Aviv University and the Helsinki Ethics Committee at Sheba Medical Center approved the study.

### Measures

Sociodemographic data, including age and sex, were collected as were the years of professional experience and the presence of physical or/and mental illness among the participants' own family members. Their perceptions of severity, certainty of diagnosis, and treatment ineffectiveness for schizophrenia/ASD/ADHD were assessed with three questions for each diagnosis on a 5-point Likert scale, where 1 represented strong disagreement with perception of the diagnosis as severe, perception of the diagnosis as a certainty, and the perception of treatment as effective, while 5 represented strong agreement (the questionnaire is provided in Supplementary Table 1).

As there are no studies written on this topic, based on clinical experience in child psychiatry (ABG) and research experience in DD (OKM), a 15-item questionnaire was developed by the research team for the purposes of this study. The questionnaire focused on negative perceptions of DD (NPDD) on a similar 5-point Likert scale as described above. The items were averaged to produce NPDD scores and reliability (schizophrenia: Cronbach's α = 0.83; ASD: Cronbach's α = 0.76; ADHD: Cronbach's α = *0.8*7), and contained statements such as “Giving a schizophrenia/ASD/ADHD diagnosis only leads to unnecessary grief among the family and child,” “There is no appropriate way to disclose the diagnosis of schizophrenia/ASD/ADHD to a family,” etc. Items that reflected a positive perception of DD (for example: “Giving a schizophrenia/ASD/ADHD diagnosis enables the family to obtain support”) were reversed (Supplementary Table 1).

Factors that potentially influenced the decision to deliver a diagnosis (FIDD) were sought by an original 6-item inventory. Those factors were independent of each other (therefore reliability was not measured for them). The participants were asked to rate the extent to which they are influenced by each factor while making the decision about delivering a certain diagnosis on a 5-point Likert scale, with 1 representing “not influencing my decision at all” and 5 representing “greatly influencing my decision”). FIDD included queries about the influence of the child's age, family's SES, parenting, and siblings (Supplementary Table 1).

An original 7-item questionnaire queried about distress accompanying DD (DADD). For each item, the participants were asked to rate the extent to which they experience distress with regard to different phases of the DD procedure on a 5-point Likert scale, where one represents no distress at all and five represents a great deal of distress. Examples are: “While informing parents about the possible symptoms of schizophrenia/ASD/ADHD,” “While recommending the transfer of the child to a special education program,” etc. Final scores and reliability (schizophrenia: α = 0.86; ASD: α = 0.77; ADHD: α = *0.8*1) were calculated based on the average rate of completed items (Supplementary Table 1).

A qualitative component using immersion/crystallization analysis method (21) included an open-ended interview focused on eliciting participants' experiences and perceptions with regard to DD of three psychiatric diagnoses: schizophrenia, ASD, and ADHD. The interview guide was designed based on former studies of DD interaction and included various invitations from participants to share their personal accounts of DD, such as the last DD, the most complicated DD, the least complicated DD, and general attitudes toward DD (5, 13). The interviews were recorded and transcribed verbatim. Two researchers (ABG and IHO) read the transcripts, after which they selected and agreed upon eight coding themes, among them recognizing DD as an emotional experience, identifying objective, and subjective factors affecting psychiatrists' level of distress accompanying DD, and recognizing whether the psychiatrist identifies with the child, parent or both. The same researchers independently coded a subsample of five participants, and the inter-rater reliability for the coding of those five participants was 0.92. The rest of the sample was coded by either one of the raters. We excluded one interview from the final analysis because of technical problems.

### Statistical Analysis

Repeated-measures analysis of variance (RM-ANOVA) was applied to assess differences in the perception of schizophrenia, ASD, and ADHD regarding each of the following measures: (a) perceived disorder severity; (b) perceived diagnosis uncertainty; (c) perceived treatment ineffectiveness; (d) negative perceptions toward DD; and (e) distress accompanying DD. Next, post hoc pairwise dependent *t*-tests were conducted to compare each pair of diagnoses (schizophrenia vs. ASD, ASD vs. ADHD, and schizophrenia vs. ADHD). Bonferroni correction for multiple comparisons was applied. Three separate linear regressions were conducted to determine the association of distress accompanying DD (as the key dependent variable) with perceived severity, uncertainty, treatment ineffectiveness, negative perceptions, items of the FIDD inventory, and demographics. To avoid multicollinearity, independent variables were decontaminated from common variance by regressing them out of each other and then inserting their standardized residuals into the final model (22). Next, the models were reduced by excluding FIDD factors that did not have significant associations [*p* < 0.016 (Bonferroni correction for multiple comparisons)] with at least one diagnosis (standardized residuals were readjusted to the final variables in the reduced model). The statistical analysis was performed with the Statistical Package for Social Sciences (SPSS), v25.0, IBM, Chicago.

## Results

The study included 80 participants, 51 of whom completed the questionnaire during the conference (72.8% participation rate) and 29 who completed the online survey (72.5%). The participants were child and adolescent psychiatrists with a mean age of 48.7 (SD = 10.7) years, and a mean of 18.6 (SD = 10.6) years of professional experience. There were 47 females (58.7%), and 21 (26.3%) of the 89 responders reported having mental illness in the family. The demographics of the study group are presented in Table 1.

**Table 1:** Sociodemographic characteristics of the study sample.

*N*	80
Age, mean (SD), in years	48.7 (10.7)
Age, range, in years	33–79
Male/Female, *n* (%)	33/47 (41.3/58.7)
Professional experience, mean (SD), in years	18.6 (10.6)
Professional experience, range, in years	2–50
Having mental illness in own family, *n* (%)	21 (26.3)

### Attitudes Toward DD of Schizophrenia, ASD, and ADHD

**Figure 1:**
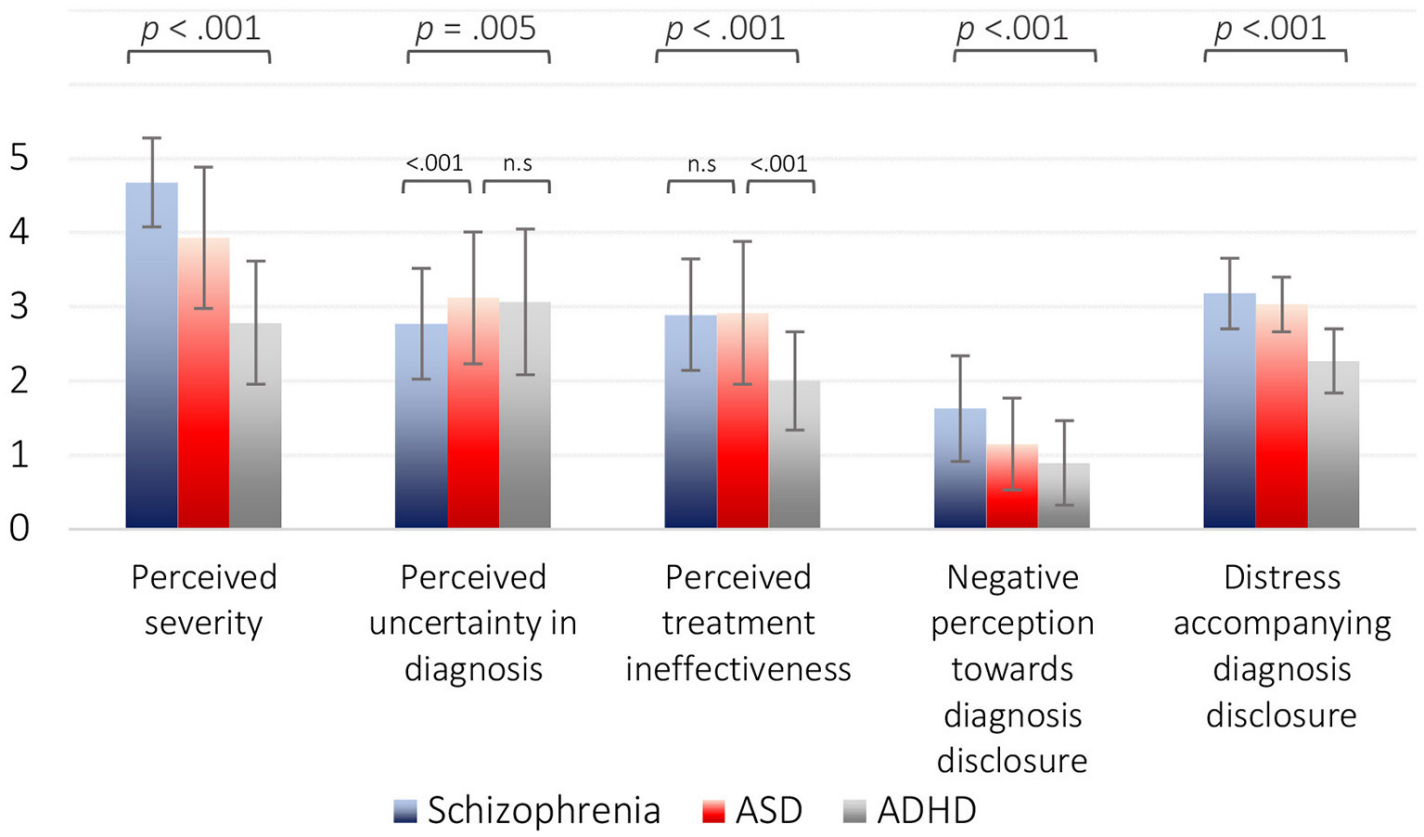
Repeated-measures ANOVA for differences across diagnoses. Perceived severity differed significantly from that of ASD and ADHD [*F*_(1,122)_ = 119.4, *p* < 0.001]. Differences in perceived uncertainty in diagnosis [*F*_(1,133)_ = 5.91, *p* = 0.005] were derived from schizophrenia being significantly lower than the other two diagnoses (*p* < 0.001). Differences in perceived treatment ineffectiveness [*F*_(1,132)_ = 46.5, *p* < 0.001] were derived from ADHD being significantly lower than ASD and ADHD (*p* < 0.001). Negative perception of diagnosis disclosure differed significantly between all three diagnoses [*F*_(1,113)_ = 111.8, *p* < 0.001]. Distress accompanying diagnosis disclosure differed significantly between all three diagnoses [*F*_(1,132)_ = 116.1, *p* < 0.001]. Greenhouse–Geisser correction was applied for all tests.

Repeated-measures ANOVA revealed that the participants perceived schizophrenia and ADHD as the most and least severe diagnoses, respectively [*F*_(1,122)_ = 119.4, *p* < 0.001]. The most negative perceptions were toward DD of schizophrenia, and the most positive were toward ADHD [*F*_(1,113)_ = 111.8, *P* < 0.001]. Distress accompanying DD was highest for schizophrenia and lowest for ADHD [*F*_(1,132)_ = 116.1, *p* < 0.001]. These results and the results of all parameters with regard to ASD are presented graphically in Figure 1.

### Stressful Factors Among Child Psychiatrists While Delivering a Diagnosis and Their Association With Experienced Distress

As presented in Table 2, the following variables were associated with distress accompanying DD in all three psychiatric diagnoses: less professional experience [schizophrenia (standardized β = −0.34, *p* < 0.001), ASD (β = −0.33, *p* = 0.003), and ADHD (β = −0.25, *p* = 0.010)], negative perception toward DD [schizophrenia (β = 0.43, *p* < 0.001), ASD (β = 0.38, *p* < 0.001), and ADHD (β = 0.40, *p* < 0.001)], the perception of the child psychiatrist of parents as being in opposition to the diagnosis [(schizophrenia: β = 0.48, *p* < 0.001; ASD: β = 0.23, *p* = 0.038, marginally significant after Bonferroni correction; ADHD: β = 0.35, *p* < 0.001)], and the presence of siblings with one of the three diagnoses in the patient's family [(schizophrenia: β = 0.46, *p* < 0.001; ASD: β = 0.28, *p* = 0.019, marginally significant; ADHD: β = 0.28, *p* = 0.009)].

**Table 2:** Linear regression with distress accompanying diagnosis disclosure as the dependent variable.

	**Distress accompanying diagnosis disclosure**					
	**Schizophrenia**		**ASD**		**ADHD**	
	**Standardized β (95% CI)**	***p*-value**	**Standardized β (95% CI)**	***p*-value**	**Standardized β (95% CI)**	***p*-value**
Sex	**0.19 (0.02, 0.53)**	**0.037**	0.02 (−0.19, 0.22)	0.878	0.06 (−0.12, 0.25)	0.508
Professional experience	**−0.34 (−0.04**, **−0.01)**	**<0.001**	**−0.33 (−0.54**, **−0.12)**	**0.003**	**−0.25 (−0.44**, **−0.06)**	**0.010**
Having mental illness in own family	0.17 (−0.01, 0.57)	0.058	0.19 (−0.02, 0.40)	0.071	**0.21 (0.03, 0.40)**	**0.026**
Negative perception toward diagnosis disclosure	**0.43 (0.19, 0.47)**	**<0.001**	**0.38 (0.16, 0.59)**	**<0.001**	**0.40 (0.20, 0.59)**	**<0.001**
Perceived morbidity severity	0.18 (0.00, 0.28)	0.054	0.08 (−0.15, 0.31)	0.467	**0.24 (0.05, 0.43)**	**0.013**
Perceived uncertainty in diagnosis	0.06 (−0.10, 0.19)	0.555	0.20 (−0.02, 0.42)	0.071	0.12 (−0.10, 0.34)	0.278
Perceived treatment ineffectiveness	0.01 (−0.13, 0.14)	0.918	−0.05 (−0.27, 0.16)	0.622	−0.03 (−0.24, 0.17)	0.751
Parents oppositional/in denial toward the diagnosis	**0.48 (0.20, 0.54)**	**<0.001**	**0.23 (0.01, 0.45)**	**0.038**	**0.35 (0.15, 0.55)**	**<0.001**
Having diagnosed sibling(s)	**0.46 (0.18, 0.52)**	**<0.001**	**0.28 (0.05, 0.51)**	**0.019**	**0.28 (0.07, 0.49)**	**0.009**

After controlling for multiple covariates, professional experience was associated with less distress accompanying DD across all diagnoses. Negative perception toward DD, parents being oppositional or in denial toward the diagnosis and having diagnosed sibling(s) were associated with more distress accompanying DD across all diagnosis.

*Bold indicates significant*.

### Qualitative Findings: Themes That Emerged From Interviews

The qualitative part of the current study involved 13 interviewees, 12 of whom completed the interview. The interviewees were all child and adolescent psychiatrists with a mean age of 46.8 (SD = 10.1) years, and an average of 16.4 (SD = 12.2) years of professional experience. Six of the interviewees were females.

The major themes that were revealed during the interviews regarding the process of DD to parents of children with schizophrenia, ASD, and ADHD were as follows:

1. DD in psychiatry is a highly charged emotional experience. Psychiatrists reported feelings of sadness, sorrow, grief, empathy, guilt, compassion, and frustration accompanying a DD. “*Before delivering the diagnosis of schizophrenia I felt tensed, my stomach was turning upside down and my heart was beating 200. It was really hard for me…;…after the delivery I felt as though I threw a bomb at the family…;…It broke my heart because I knew how much they didn't want such a life for their daughter…;…I couldn't stop thinking about it while at home… although it was a long time ago it was so powerful that I recall it as if it was yesterday…*”
2. DD of psychiatric disorders is a continuous process and not a time-limited task. “*I find myself delivering the diagnosis in steps, adjusting the delivery to what I believe the family is capable of hearing…;…sometimes people don't want to hear the news at first…;…they sit in front of me and listen but it seems that nothing gets through…;…sometimes the process of understanding and accepting takes time…*”
3. The challenge in preserving hope. “*I often deal with the ethical question if our role is to stick to the truth or to preserve hope…I feel that my role is to soften the bad news*… “*In my office I hang a picture of my only patient with a syndrome who got married…;…I never say out loud things that close the door to hope.we must preserve hope not only for our patients but also for ourselves…;…our hope enables us to continue to search for treatment*”
4. During the psychiatric evaluation, a child psychiatrist tends to identify either with the child or with the parent and those choices affect the DD. Psychiatrists reported that their identification and overinvolvement with either child or parent affects their DD so that the process becomes more emotional. Younger psychiatrists reported that they tend to identify with the child and that they feel obligated to deliver the diagnosis so that the child will receive the appropriate treatment. (“*I believe that my main role is to represent the child's best interests*”), while older psychiatrists reported that they tended to identify with the parents and felt obligated to deliver the diagnosis in a vague and optimistic way (“*I try to deliver DD in an ambiguous way. It's good both for me and for the parents. For example, I might say to parents that their child has high functioning autism and that in the past children like him were not meeting the diagnostic criteria for autism…;…that there is a good chance that their child will succeed in life…;…I always keep in mind that children are their parents' greatest hope and that you need a good reason to take that hope away*”).
5. Psychiatrists revealed different attitudes toward the legal principle endorsing the position of “the patient's right to know.” Overall, younger psychiatrists believed that DD should be shared under any circumstances. “*I share the diagnosis as soon as I know it because I believe it's the patient's and the parents' right to know*.” Conversely, older psychiatrists believed that DD of severe psychiatric disorders should be delivered only under certain circumstances. “*I feel that to deliver a parent their child's psychiatric diagnosis is the biggest responsibility ever. I do not share the worst thoughts that run in my head with parents and I do not use the DSM vocabulary. I express only what I think people are capable of hearing and what I assume would be helpful for them. For many years I avoided saying the diagnosis of* “*schizophrenia*” *to parents and it is still hard for me to do so…; …sometimes I ask myself what is the purpose of giving a diagnosis that has poor long-acting outcomes…;…treatment is more important than the diagnosis*.”
6. Psychiatrists, at all ages, shared that if it was their child who was psychiatrically ill, they would have preferred to receive a clear, direct, and honest DD. “*I would want to know everything regarding the diagnosis of my own child, but this is me, it is not true for everyone…not everyone wants to know everything*…”
7. Main objective and subjective factors that affect psychiatrists' level of distress accompanying the DD of psychiatric disorders:
  - The perception of the degree of coping and the strength and resilience of families.
    “*When I feel that the family has the strength to deal with the diagnosis of ASD and that the child is in good hands, I become optimistic and the process of DD becomes simple*”
  - The perception of the family's readiness and willingness to receive a diagnosis “*It's easy to deliver a diagnosis to families who are willing to accept it… …I recall a situation that I disclosed a diagnosis of schizophrenia to a family that was not ready to cope with it and they never showed up again*”
  - Family situations where another child has already been diagnosed with a psychiatric disorder were reported as a burden when considering DD. “*How can I tell parents that their second child is also suffering from ASD? What will they think to themselves? Why them again?*”
  - Psychiatrist's own perception regarding the certainty of the different psychiatric disorders. “*When I deliver the diagnosis of ADHD it doesn't feel certain… …I think that there are not enough scientific data behind it. It is not equivalent to DD of cancer*.” “*DD of schizophrenia sometimes makes me feel guilty for shattering someone's dream when I am not even sure that I am right*”
8. The feelings of loneliness in the DD and the wish to share and reflect. “*Agreement with other colleagues on the diagnosis and the company of another colleague in the process of DD itself provides support and increases my confidence*.” “*I feel that the process of sharing always helps me…;..this interview made me raise questions and assisted me by providing an opportunity to reflect on this issue of DD. I wish there were more professional forums to talk and consult about the DD. I wish I had more opportunities to discuss this in my everyday life*.”

## Discussion

This study explored attitudes toward DD and possible stressful factors in the process of DD among child psychiatrists. The findings suggest that attitudes varied according to diagnosis and that there are multiple stressful factors while delivering a diagnosis in general, as well as unique factors that are related to a specific diagnosis. The facts that the diagnosis of schizophrenia was perceived as severe and that there is no effective treatment were found to be associated with negative attitudes toward DD. This was supported by both quantitative and qualitative data. The qualitative data revealed the challenges and dilemmas and suggested additional factors that affect child psychiatrists' attitudes toward DD: their perception concerning the legal obligation to deliver a diagnosis, their identification either with the child or with the parent, their perceptions of the parents' willingness and ability to cope with the news, and the level of devastation expected to be experienced.

The diagnosis of ASD was perceived by child psychiatrists as less severe than the diagnosis of schizophrenia, and they reported having less-negative attitudes toward DD of ASD and experiencing less stress accompanying the DD compared to that of schizophrenia. The finding that DD of ASD is perceived by child psychiatrists as less negative and stressful is interesting and may be explained by the following. In the past, both schizophrenia and ASD were considered a single clinical entity termed “childhood schizophrenia” (23). Even though the two diagnoses were subsequently separated in the DSM-III, both were considered severe disorders until the last two decades. We believe that ASD was gradually considered as a less severe disorder due to lowering the threshold of diagnosis. For example, currently the intelligence of most children that are diagnosed with ASD is within the normal range whereas in the past most of the children with the diagnosis of ASD were diagnosed also with intellectual disability (24). In addition, in Israel and in other western countries, the diagnosis of ASD enables families to receive considerable support, including treatments (e.g., applied behavioral analysis, occupational and speech therapy) and one-on-one school assistance. Thus, it can be assumed that while delivering the diagnosis of ASD, child psychiatrists perceive not only the negative implications of the diagnosis on various life domains but also the benefits of increased support that the DD of ASD will provide to the child and family.

The findings on attitudes toward DD emphasize the important effect of illness perception on the DD process. Extensive research has addressed the effects of psychiatric illness perception on patients and relatives (25, 26), showing that negative perception, mostly in the form of stigma against psychiatric disorders, interferes with an adaptive coping process [see review of (14), on parents' perceptions]. Psychiatric illness perception of professionals, however, is barely studied, probably assuming that their perception is relatively objective and less prone to biases or that, as professionals, they are less prone to be influenced by their biases. However, there is evidence that psychiatrists tend to perceive serious mental illness as schizophrenia as “bombastic” in its implications or as “being sick in mind,” and ASD in child psychiatry as devastating (27), which are perceptions that were identified as likely to create reluctance to DD to patients and families (13). The findings of the current study further support those conclusions by showing an association between the perception of a given mental illness as being severe, the diagnosis as being uncertain, and the treatment as being ineffective, with negative attitudes toward DD among child psychiatrists.

The distress psychiatrists experience while practicing DD and its correlates is an additional major finding in the current study. Less professional experience, negative attitudes toward DD, and the perception that the parents opposed the diagnosis were all associated with higher DD-related stress. In line with these questionnaire responses, the qualitative analysis also showed that psychiatrists experience various negative feelings, such as sadness, sorrow, grief, empathy, guilt, compassion, and frustration while delivering a diagnosis, and even long after. This led to their wish for support and for opportunities to share and reflect with colleagues on these challenges in preparation to and following these encounters.

Our finding that psychiatrists with fewer years of professional experience reported more stress accompanying DD may be explained by the fact that DD requires high communication skills that are usually achieved over time. In addition, younger psychiatrists reported feeling obligated to deliver the diagnosis for the benefit of the child more than older psychiatrists. This feeling of obligation may limit younger psychiatrists' freedom to choose whether to delay or soften the DD and, in turn, may also explain their experience of DD as more stressful.

As had been shown in previous studies in psychiatry [see review of (16)] as well as in the present one, the process of DD included psychosocial aspects that emerged from the connection with the family and the psychiatrist's perception of the way the parents cope with the verdict. Further exploration is needed to understand the source of these perceptions and their accuracy, and perhaps find ways to help psychiatrists enhance families' resilience and willingness to accept the disease.

There are several limitations to this study that bear mention. First, it focused on DD of three diagnoses, thereby limiting generalization of the findings to other psychiatric diagnoses. Second, the study design was cross-sectional and therefore it is not clear how perception and attitudes affect the experienced distress longitudinally. Lastly, the study was conducted from the perspectives of psychiatrists and did not address the perspectives of patients and relatives. Perkins et al. (16) recently recommended a triangular approach to the understating of DD in psychiatry, which includes psychiatrists, patients, and relatives, and future studies should combine them to better explore the more inclusive dynamics of DD.

The current study highlighted the importance of perceptions and attitudes of psychiatrists in the process of DD. The findings suggest that child psychiatrists are affected by their own perception of disorders and of DD as well as by additional factors that are operative in the interpersonal domain of communicating DD. We believe that these results can assist in tailoring interventions to assist psychiatrists in coping with DD of conditions that have far-reaching ramifications as well as with their own biases. This, in turn, may improve the way DD is practiced, leading to a better experience for the psychiatrist, the patient, and the family.

## Data Availability Statement

The raw data supporting the conclusions of this article will be made available by the authors, without undue reservation.

## Ethics Statement

The studies involving human participants were reviewed and approved by Tel Aviv University Institutional Review Board and Sheba Medical Center Institutional Review Board. The patients/participants provided their written informed consent to participate in this study.

## Author Contributions

AB-G, DG, and OK-M: conceiving and designing the study. AB-G, and DB: data collection. NH-P: statistical analyses. AB-G, IH-O: qualitative analyses. AB-G, IH-O, NH-P, DG, and OK-M: data interpretation. AB-G, IH-O, NH-P, DG, and OK-M: writing of the final manuscript. All authors contributed to, reviewed, and approved the final manuscript.

## Conflict of Interest

The authors declare that the research was conducted in the absence of any commercial or financial relationships that could be construed as a potential conflict of interest.
