# Supplementary material for: The Delivery of Diagnosis by Child Psychiatrists: Process Characteristics and Correlates of Distress
Source: Front Psychiatry. 2021 Mar 22;12:632207. doi: 10.3389/fpsyt.2021.632207 (PMC8019702; doi:10.3389/fpsyt.2021.632207)
Supplement: Supplementary file 1 [file Data_Sheet_1.docx]

**Supplements**

**Translation process**

The questionnaires were originally written in Hebrew and were translated to English by a bilingual Hebrew and English Speaker. Next, questionnaires were back translated to Hebrew by a second bilingual speaker who was blinded to the original version. Each back-translated item was then compared to the original item in Hebrew, and if the two versions differed significantly the process was repeated until complete agreement was reached.

**Questionnaires:**

Please read the following statements and rate the extent to which you agree with each from 1-5, where 1 represents strong disagreement, 3 represents neutrality or uncertainty towards the statement, and 5 represents complete strong agreement.

| **5- Strongly agree** | **4-**  **Agree** | **3-**  **Unsure** | **2-**  **Disagree** | **1- Strongly disagree** | **Item** |
| --- | --- | --- | --- | --- | --- |
|  |  |  |  |  | Schizophrenia is a severe disorder |
|  |  |  |  |  | The diagnosis of Schizophrenia is clear-cut |
|  |  |  |  |  | Treatment of Schizophrenia is effective |
|  |  |  |  |  | Giving a diagnosis of Schizophrenias reduces uncertainty in the family and enables the family to obtain medical information * |
|  |  |  |  |  | Giving a diagnosis of Schizophrenia enables the family to engage in open discussion and escape isolation * |
|  |  |  |  |  | Giving a diagnosis of Schizophrenia enables the family to obtain support * |
|  |  |  |  |  | Parents are interested to know if their child has the diagnosis of Schizophrenia * |
|  |  |  |  |  | Giving a diagnosis of Schizophrenia reduces feelings of guilt among family members * |
|  |  |  |  |  | Giving a diagnosis of Schizophrenia enhances trust and communication between the family and physician * |
|  |  |  |  |  | Giving a diagnosis of Schizophrenia might disrupt both the family’s and child’s self-image |
|  |  |  |  |  | Giving a diagnosis of Schizophrenia leads to unnecessary grief among the family and child |
|  |  |  |  |  | Giving a diagnosis of Schizophrenia might disrupt the family’s relationship with the physician |
|  |  |  |  |  | Giving a diagnosis of Schizophrenia might diminish the family’s and child’s hope |
|  |  |  |  |  | Giving a diagnosis of Schizophrenia may lessen the family’s and child’s cooperation with therapy |
|  |  |  |  |  | Family members’ emotional responses during diagnosis disclosure of Schizophrenia are harsh and unpredictable |
|  |  |  |  |  | When the parents are struggling with other difficulties, it is an inappropriate time to disclose the diagnosis of Schizophrenia |
|  |  |  |  |  | There is no appropriate way to disclose the diagnosis of Schizophrenia to a family |
|  |  |  |  |  | I avoid disclosing the diagnosis of Schizophrenia to families |

* Reverse items are marked with an asterisk.

Please read the following statements and rate the extent to which each of these factors **influence your decision to give a Schizophrenia diagnosis** from 1-5, where 1 represents ‘not influencing my decision at all’, 3 represents neutrality or uncertainty towards the statement, and 5 represents ‘substantially influences my decision’.

| **5- a great deal** | **4** | **3** | **2** | **1- Not at all** | **Item** |
| --- | --- | --- | --- | --- | --- |
|  |  |  |  |  | Child’s young age |
|  |  |  |  |  | Supportive and stable family |
|  |  |  |  |  | Low socioeconomic status |
|  |  |  |  |  | Parental opposition or in denial towards the diagnosis |
|  |  |  |  |  | Parents with difficulty in understanding |
|  |  |  |  |  | Presence of siblings |
|  |  |  |  |  | Presence of diagnosed sibling(s) |

Please read the following statements and rate the **level of distress** that you experience regarding each statement, from 1-5, where 1 represents no distress at all, 3 represents neutrality or uncertainty towards the statement, and 5 represents a great deal of distress.

| **5- A great deal** | **4** | **3** | **2** | **1- Not at all** | **Item** |
| --- | --- | --- | --- | --- | --- |
|  |  |  |  |  | Before disclosing the diagnosis of Schizophrenia |
|  |  |  |  |  | While giving information to the parents about symptoms of Schizophrenia |
|  |  |  |  |  | While giving information to the parents about the future implications of Schizophrenia on the child and the family |
|  |  |  |  |  | While recommending medications |
|  |  |  |  |  | While recommending transferring the child into a special education program |
|  |  |  |  |  | While recommending hospitalization |
|  |  |  |  |  | After disclosing the diagnosis of Schizophrenia |

Please read the following statements and rate the extent to which you agree with each from 1-5, where 1 represents strong disagreement, 3 represents neutrality or uncertainty towards the statement, and 5 represents complete strong agreement.

| **5- Strongly agree** | **4** | **3** | **2** | **1- Strongly disagree** | **Item** |
| --- | --- | --- | --- | --- | --- |
|  |  |  |  |  | ASD is a severe disorder |
|  |  |  |  |  | The diagnosis of ASD is clear-cut |
|  |  |  |  |  | Treatment of ASD is effective |
|  |  |  |  |  | Giving an ASD diagnosis reduces uncertainty in the family and enables the family to obtain medical information * |
|  |  |  |  |  | Giving an ASD diagnosis enables the family to engage in open discussion and escape isolation * |
|  |  |  |  |  | Giving an ASD diagnosis enables the family to obtain support * |
|  |  |  |  |  | Parents are interested to know if their child has the diagnosis of ASD * |
|  |  |  |  |  | Giving an ASD diagnosis reduces feelings of guilt among family members * |
|  |  |  |  |  | Giving an ASD diagnosis enhances trust and communication between the family and physician * |
|  |  |  |  |  | Giving an ASD diagnosis might disrupt both the family’s and child’s self-image |
|  |  |  |  |  | Giving an ASD diagnosis leads to unnecessary grief among the family and child |
|  |  |  |  |  | Giving an ASD diagnosis might disrupt the family’s relationship with the physician |
|  |  |  |  |  | Giving an ASD diagnosis might diminish the family’s and child’s hope |
|  |  |  |  |  | Giving an ASD diagnosis may lessen the family’s and child’s cooperation with therapy |
|  |  |  |  |  | Family members’ emotional responses during diagnosis disclosure of ASD are harsh and unpredictable |
|  |  |  |  |  | When the parents are struggling with other difficulties, it is an inappropriate time to disclose the diagnosis of ASD |
|  |  |  |  |  | There is no appropriate way to disclose the diagnosis of ASD to a family |
|  |  |  |  |  | I avoid disclosing the diagnosis of ASD to families |

* Reverse items are marked with an asterisk.

Please read the following statements and rate the extent to which each of these factors **influence your decision to give an ASD diagnosis** from 1-5, where 1 represents ‘not influencing my decision at all’, 3 represents neutrality or uncertainty towards the statement, and 5 represents ‘substantially influences my decision’.

| **5- a great deal** | **4** | **3** | **2** | **1- Not at all** | **Item** |
| --- | --- | --- | --- | --- | --- |
|  |  |  |  |  | Child’s young age |
|  |  |  |  |  | Supportive and stable family |
|  |  |  |  |  | Low socioeconomic status |
|  |  |  |  |  | Parental opposition or in denial towards the diagnosis |
|  |  |  |  |  | Parents with difficulty in understanding |
|  |  |  |  |  | Presence of siblings |
|  |  |  |  |  | Presence of diagnosed sibling(s) |

Please read the following statements and rate the **level of distress** that you experience regarding each statement, from 1-5, where 1 represents no distress at all, 3 represents neutrality or uncertainty towards the statement, and 5 represents a great deal of distress.

| **5- A great deal** | **4** | **3** | **2** | **1- Not at all** | **Item** |
| --- | --- | --- | --- | --- | --- |
|  |  |  |  |  | Before disclosing the diagnosis of ASD |
|  |  |  |  |  | While giving information to the parents about symptoms of ASD |
|  |  |  |  |  | While giving information to the parents about the future implications of ASD on the child and the family |
|  |  |  |  |  | While recommending medications |
|  |  |  |  |  | While recommending transferring the child into a special education program |
|  |  |  |  |  | While recommending hospitalization |
|  |  |  |  |  | After disclosing the diagnosis of ASD |

Please read the following statements and rate the extent to which you agree with each from 1-5, where 1 represents strong disagreement, 3 represents neutrality or uncertainty towards the statement, and 5 represents complete strong agreement.

| **5- Strongly agree** | **4** | **3** | **2** | **1- Strongly disagree** | **Item** |
| --- | --- | --- | --- | --- | --- |
|  |  |  |  |  | ADHD is a severe disorder |
|  |  |  |  |  | The diagnosis of ADHD is clear-cut |
|  |  |  |  |  | Treatment of ADHD is effective |
|  |  |  |  |  | Giving an ADHD diagnosis reduces uncertainty in the family and enables the family to obtain medical information * |
|  |  |  |  |  | Giving an ADHD diagnosis enables the family to engage in open discussion and escape isolation * |
|  |  |  |  |  | Giving an ADHD diagnosis enables the family to obtain support * |
|  |  |  |  |  | Parents are interested to know if their child has the diagnosis of ADHD * |
|  |  |  |  |  | Giving an ADHD diagnosis reduces feelings of guilt among family members * |
|  |  |  |  |  | Giving an ADHD diagnosis enhances trust and communication between the family and physician * |
|  |  |  |  |  | Giving an ADHD diagnosis might disrupt both the family’s and child’s self-image |
|  |  |  |  |  | Giving an ADHD diagnosis leads to unnecessary grief among the family and child |
|  |  |  |  |  | Giving an ADHD diagnosis might disrupt the family’s relationship with the physician |
|  |  |  |  |  | Giving an ADHD diagnosis might diminish the family’s and child’s hope |
|  |  |  |  |  | Giving an ADHD diagnosis may lessen the family’s and child’s cooperation with therapy |
|  |  |  |  |  | Family members’ emotional responses during diagnosis disclosure of ADHD are harsh and unpredictable |
|  |  |  |  |  | When the parents are struggling with other difficulties, it is an inappropriate time to disclose the diagnosis of ADHD |
|  |  |  |  |  | There is no appropriate way to disclose the diagnosis of ADHD to a family |
|  |  |  |  |  | I avoid disclosing the diagnosis of ADHD to families |

* Reverse items are marked with an asterisk.

Please read the following statements and rate the extent to which each of these factors **influence your decision to give an ADHD diagnosis** from 1-5, where 1 represents ‘not influencing my decision at all’, 3 represents neutrality or uncertainty towards the statement, and 5 represents ‘substantially influences my decision’.

| **5- a great deal** | **4** | **3** | **2** | **1- Not at all** | **Item** |
| --- | --- | --- | --- | --- | --- |
|  |  |  |  |  | Child’s young age |
|  |  |  |  |  | Supportive and stable family |
|  |  |  |  |  | Low socioeconomic status |
|  |  |  |  |  | Parental opposition or in denial towards the diagnosis |
|  |  |  |  |  | Parents with difficulty in understanding |
|  |  |  |  |  | Presence of siblings |
|  |  |  |  |  | Presence of diagnosed sibling(s) |

Please read the following statements and rate the **level of distress** that you experience regarding each statement, from 1-5, where 1 represents no distress at all, 3 represents neutrality or uncertainty towards the statement, and 5 represents a great deal of distress.

| **5- A great deal** | **4** | **3** | **2** | **1- Not at all** | **Item** |
| --- | --- | --- | --- | --- | --- |
|  |  |  |  |  | Before disclosing the diagnosis of ADHD |
|  |  |  |  |  | While giving information to the parents about symptoms of ADHD |
|  |  |  |  |  | While giving information to the parents about the future implications of ADHD on the child and the family |
|  |  |  |  |  | While recommending medications |
|  |  |  |  |  | While recommending transferring the child into a special education program |
|  |  |  |  |  | While recommending hospitalization |
|  |  |  |  |  | After disclosing the diagnosis of ADHD |
